# Scaling up resource recovery from wastewater: A socio-technical perspective

**DOI:** 10.1016/j.ese.2026.100734

**Published:** 2026-07-16

**Authors:** Bernhard Truffer, Christian Binz, Eberhard Morgenroth

**Affiliations:** a Eawag – Swiss Federal Institute for Aquatic Science and Technology, Dübendorf, Switzerland; b Copernicus Institute of Sustainable Development, Utrecht University, the Netherlands; c CIRCLE - Centre for Innovation Research at Lund University, Lund, Sweden; d ETH- Swiss Federal Institute of Technology, Zürich, Switzerland

**Keywords:** Resource recovery, Wastewater, Socio-technical transitions, Utilities, Innovation

## Abstract

Resource recovery has gained substantial traction in urban water management in recent years, promising exciting opportunities for utilities to develop novel services, new income streams, and/or expand the environmental benefits of their operations. However, success stories that substantiate the high hopes remain scant. We will argue that a transition of the sanitation sector from providing a public good of waste disposal to running a resource mining venture will require a systemic innovation process in which technical, organizational, and regulatory routines of involved actors will be deeply reshuffled. We review the state of knowledge on the opportunities and barriers for scaling up innovative resource recovery solutions from wastewater and illustrate them with three short case studies of existing resource recovery systems. While the complexity of inducing resource recovery should not be underestimated, transformative innovation trajectories can be successful if technological, social, and organizational challenges are strategically and holistically addressed.

## Highlights

• Resource recovery from wastewater is increasingly getting attention in research and practice.
• Supplementary innovations in regulatory, organizational and market dimensions are often overlooked.
• A socio-technical perspective on these multi-faceted innovation processes is necessary.
• Water utilities need new capabilities to engage in these innovation processes.

## 1 Promises and challenges of resource recovery from wastewater

One of the most fundamental shifts in the perception of wastewater is that it is increasingly seen as a trove of untapped resources instead of a waste stream that needs “cleaning”. At the turn of the 20th century, the wastewater sector emerged to address pressing public hygiene problems in urban centers. By the middle of the century, its mandate extended towards environmental protection. The technologies and social structures that provide urban hygiene and environmental protection have developed over more than 100 years and are now highly standardized and optimized to deliver these tasks [1]. Turning toward understanding wastewater as a mineable resource stock will require not only new technologies, but also new mindsets, organizational and regulatory capabilities, and routines [2]. We therefore argue that identifying crucial success conditions for such a transition asks for an integrated “socio-technical” perspective that focuses on the interplay between the needed technical and social innovations [3].

Resource recovery from wastewater is by no means a radically new approach. Fecal matter has been used as a fertilizer since ancient times. Yet, due to concerns about organic pollutants and heavy metals, land application of biosolids (e.g., by removing excess sludge from wastewater treatment) in agriculture has increasingly been considered too risky. Instead, other valuable resources in wastewater have come into focus [4] (Table 1). A first, rather obvious recovery option is recycling the water itself. With today's technologies, wastewater can be treated to drinking water quality and reused directly or indirectly in the urban water cycle. Second, wastewater contains substantial amounts of energy that can be recovered for heating purposes or organic matter that may be transformed into electricity through biogas. Third, nutrients can be transformed into fertilizer if appropriately treated and packaged. More recently, extraction of chemicals (e.g., biopolymers), cellulose fibers, or precious metals (e.g., gold, lithium) has been proposed.

**Table 1:** Examples of valuable compounds and possible products that can be produced from wastewater ^[based on^ [4]^,^ [5]^,^ [6]^]^.

Type of resource	Possible products
Water	Potable water (direct or indirect potable reuse) Non-potable water for urban reuse (e.g., toilet flushing or urban irrigation) Non-potable water for agricultural irrigation Industrial water reuse (recycling within industry or municipal wastewater treated for reuse in industry)
Nutrients	Phosphorus Nitrogen Potassium Overall nutrients
Energy	Heat Electricity
Organics	Methane Volatile fatty acids Bioplastics (e.g., polyhydroxyalkanoates [PHA]) Cellulose Dyes
Metals and other inorganics	Precious metals (e.g., gold, lithium) Copper, nickel, zinc Aluminum and iron hydroxides Salts

Tapping into recovering and selling these resources requires manifold innovation processes by the operating utilities, which often transcend their established routines and organizational capabilities. We appreciate that substantial progress has been achieved in research and development in terms of identifying potentially valuable resources and developing technologies to extract and valorize them. However, the organizational and regulatory implications have received far less proactive engagement in research and practice.

What do we know about the success conditions of such a transition, and what are realistic expectations about how a transformation of the wastewater sector might come about? This is the key question of this perspective paper. We will first present a rough characterization of challenges and opportunities that exist in the different resource streams that could potentially be tapped from wastewater. Then we will elaborate on key organizational and regulatory challenges that are associated with this transition. In section 4, we will provide three illustrative examples of how socio-technical transitions have started to shape up. Finally, we conclude by outlining strategic implications for utilities, technology companies, professional associations, researchers, and regulators seeking to actively support the broader mainstreaming of resource recovery solutions.

## 2 The rapidly expanding strategic options space of resource recovery

Wastewater contains a range of valuable materials for reuse. With 360 billion m^3^ of wastewater generated globally every year, it carries enormous amounts of resources [7]. The problem is that many of these resources are diluted and mixed in a complex wastewater matrix. Extracting, concentrating, and purifying these materials at sufficient scale thus implies significant effort and cost. Relevant costs may be monetary, but also include energy demand, chemical demand, or emissions.

Whether or not a resource is in fact sufficiently valuable for recovery depends strongly on the local context. There are five main factors that, depending on context, could determine whether sufficient value can be extracted from resources available in wastewater. We will illustrate them using water reuse as an example.

***Factor 1: Scarcity*.** When conventional water supplies can no longer meet demand, reusing treated wastewater allows the city to overcome scarcity. This is why an increasing number of cities worldwide have opted for potable water reuse, including Windhoek, Singapore, Los Angeles, San Diego, and Sydney, or non-potable reuse, including Tokyo, San Francisco, Bengaluru, and Barcelona. These cities cannot continue business as usual without running out of water.

***Factor 2: Derisking*.** Many fast-growing cities today may not be literally running out of water. But they are still putting in place governance and are implementing technology for water reuse as a precautionary measure to be prepared for the future. It is simply too risky to hope that business as usual will provide sufficient water in light of population growth and climate variability. Providing a range of conventional and unconventional water supply options helps to prepare for an uncertain future. Policies asking for a diversification of available water sources have gained traction in cities around the world.

***Factor 3: Opportunity and prestige.*** Technology for water reuse is becoming cheaper, more robust, and more user-friendly. In cities with high water prices, it is already becoming economically beneficial to reuse water. Such water reuse can be at the building scale or from centralized wastewater treatment plants. Being the first place to successfully apply an innovative approach can come with considerable prestige in the sector. End users’ increasing willingness to pay for advanced water reuse systems also provides an opportunity to make water management more circular.

***Factor 4: Positive spillovers*.** Requirements for wastewater treatment are demanding increasingly higher quality. For example, Switzerland and now also the European Union (EU) are requiring not only organic carbon and nutrient removal but also the removal of organic micropollutants (quaternary treatment). With the requirements for environmental discharge increasing, it becomes increasingly easier to do the extra treatment and produce water that is suitable for water reuse.

***Factor 5: Overall environmental benefits*.** Sustainable recovery of products from wastewater must consider the overall benefits of products, overall emissions and contamination, and overall costs. Such products will always contain some degree of contamination, and there will always be additional emissions or demand for chemicals and energy. Again, in the case of water reuse, the environmental trade-offs may include contaminants in the produced water, increased energy demand and greenhouse gas emissions, and waste products such as brine (in the case of reverse osmosis treatment) that must be managed. Whether or not recovery provides overall environmental benefits depends on the local context.

Besides water reuse, phosphorus recovery from wastewater has received increasing attention as it is a limiting resource already today (Factor 1) or may become limited in the future (Factor 2) [8]. Using Switzerland as an example, countrywide phosphorus recovery was triggered by the environmental protection law that requires the recovery of phosphorus from sewage sludge (https://www.bafu.admin.ch/en/phosphorrecycling-en). Currently, waste sludge is mono-incinerated, and ash is stored in dedicated places to provide a phosphorus repository for the future. Even though there is no immediate phosphorus scarcity and there is currently also not sufficient opportunity to recover the phosphorus for agriculture, the current approach is mostly driven by the derisking factor. But nutrient recovery in Switzerland could also be an opportunity. The start-up company Vuna produces Aurin, a fertilizer derived from human urine collected and treated in decentralized systems. The company can make a profit selling this fertilizer as people are willing to pay a premium for this innovative approach of closing nutrient cycles.

## 3 What would it take to seize resource recovery opportunities?

We can derive from this short elaboration that there is a need to know more about mineable resources in wastewater and suitable technologies to extract them. But importantly, we also see that venturing into any of these innovations will require that utilities and other key actors in the sanitation sector change their modes of operation. Given that wastewater utilities were historically optimized for cleaning up cities from polluted waters, they will have to learn how to engage in new markets (basic chemicals, energy provision, water supply, etc.), to build up new competences (targeting new customer segments, developing new logistics and value chains, etc.) and to change their basic operating culture—for example, by attracting new types of personnel with unconventional competencies Kiparsky, Sedlak [9]. What do we know from social science literature on how organizations successfully change for new businesses that deviate substantially from their established competences?

In management studies, it is well established that incumbent organizations in any sector are not comfortable with innovations that do not easily align with their existing capabilities [10]. Radically new products or processes often challenge established ways of working, pushing organizations to change their routines, sometimes even to the point of jeopardizing established business lines. This may lead to stranded investments, which for utilities in the sanitation sector is particularly daunting due to its very long-lasting technical components (treatment plants, sewer networks, etc.). Furthermore, it is quite well established that wastewater utilities suffer from an innovation deficit [9] mostly because of their narrow and highly regulated mandates and very limited incentives for opening new income streams. In consequence, innovations are often developed in expert networks between utilities, industry associations, and technical universities. While this is a rational organizational setup for the current core mission, it may entrench the sector in pursuing well-established technologies for too long.

The literature on sustainability transition studies has shown that this represents a rather typical situation [11]. The automotive industry in the United States and Germany sticking with combustion engines for too long offers a vivid example of the adverse outcomes of such lock-ins. A vast body of evidence shows that a key cause of such lock-ins is that internal company structures are often insufficient to realize radical innovation [12]. Radical innovation requires overcoming manifold interrelated barriers like lacking appropriate knowledge, the need for establishing new markets, mobilizing critical financial resources, setting new technological standards, developing dominant designs for the new technology, or achieving widespread legitimacy for the new products and services [[13], [14], [15], [16]]. This becomes all the more challenging if these newly established markets may cross different economic sectors, which is the case for many of the resource recovery options discussed above.

These barriers are interrelated, reinforcing each other. They may only be overcome if different actors with complementary organizational resources form so-called technological innovation systems (TIS), which are coordinated by professional associations, applied research organizations, non-governmental organizations, or large companies [13,14]. In these systems, actors jointly work on building up the technological skills, standards, narratives, and financial resources that are necessary for scaling up the new technologies. Given the complexity of system building processes and substantial uncertainties that come with radical innovations, innovators are often bound to tinker with a wide range of potential alternatives and run experiments in real-world settings to identify promising options and learn about how to address system barriers [17,18]. An illustrative case is the rise of solar photovoltaics, which moved from an overly expensive and inefficient niche product in the 1970s to the most cost-competitive option for electricity generation in 2025 [19]. This change is due to impressive technological development, but maybe even more importantly, to innovation in manufacturing, business models, regulations, user behavior, etc. [20,21].

What does this mean for wastewater utilities? As innovation management in the water sector is today often organized through associations that coordinate TIS-like actor networks, engaging in a new TIS would not represent a radically new mode of operation for many utilities. However, given that each resource recovery option hinges on very different systemic barriers and potential markets, utilities might be overwhelmed as they have been optimized to deliver services cheaply and reliably, and often lack the financial means to build up innovation management departments. They also typically have little direct exposure to the needs of customers, which proves challenging when introducing innovations that suffer from legitimacy deficits and are therefore received with skepticism by the relevant customer segments [16].

Utilities also typically have a workforce dominated by technical engineers with high skills in established technologies and the maintenance of existing infrastructures. This often leads to risk-averse mindsets, as disruptions in the core business have dire consequences for entire cities or regions. Financially, their business operations are oriented at fixed renumerations for public services to a region for which utilities hold a regulated service monopoly. On the technology cost side, utilities are used to consider economies of scale at the level of the treatment reactor (e.g., the treatment plant), which provides strong incentives for centralization, while many resource-related innovations would profit from economies of the number of units produced, which often drives cost decreases for decentralized technologies [[22], [23], [24]].

Engaging in resource recovery initiatives hence requires deep transformations of utilities’ modus operandi compared to past innovations. However, the exciting opportunity with resource recovery options is that a wide variety of new activities and inspiring business opportunities could develop in the future. This may attract young people from diverse backgrounds and position utilities as exciting and innovative places to work in. In addition, venturing into resource recovery may turn utilities into service providers with a broader income base from which future core operations and innovation activities may be funded.

## 4 Illustrative cases of places that have successfully transitioned into resource recovery

We will briefly characterize three well-documented cases of innovative resource recovery solutions, which illustrate the technical and organizational challenges that come with inducing transformative innovation in the sanitation sector.

(1) Distributed non-potable water reuse (Tokyo, Japan; New York and San Francisco, the United States; Beijing, China; Bengaluru, India; and Barcelona, Spain)

Cities that install distributed water reuse systems, which treat wastewater locally and reuse it for non-potable purposes, have been mushrooming internationally over the past decades [25]. Major factors driving this development have been scarcity, derisking, and technological opportunities, which, however, also come with strong environmental trade-offs. For instance, Bengaluru is one of the most rapidly growing urban centers in the world, which is confronted with very severe water scarcity problems. As a pragmatic response to dealing with these challenges and for de-risking against future drought problems, more than 4000 decentralized wastewater treatment plants have been installed across the city.

Building up the organizational capabilities to manage such a distributed fleet of treatment systems, however, exceeds the core capabilities of any wastewater utility. The local utility has, therefore, for a long time, ignored this rapidly growing market, which has been captured by an emerging TIS of local technology providers, consultants, research institutes, etc. [25]. Only very recent developments indicate that the utility is venturing into a new role as an ecosystem orchestrator. This role would enable the utility to build on its strengths without having to deeply engage in the operation and maintenance of the plants. In San Francisco, the utility has been more proactive by forming a dedicated team of experts with the necessary management and leadership skills needed for this orchestration task [26]. In Bengaluru and Beijing, the utility has either not engaged enough or tried to control distributed systems too much, both of which have led to widespread system failures [27].

(2) District-scale nutrient and energy recovery (Sneek, the Netherlands; Hamburg, Germany; Helsingborg, Sweden; and Ghent, Belgium)

A second, quickly expanding model is district-scale resource recovery systems that aim to maximize nutrient and energy recovery. Driven by a de-risking strategy, as well as by technological opportunities and ambitious local policies asking new developments to reach NetZero greenhouse gas emissions, utilities in Northern Europe have coined an innovative water management approach that uses vacuum pipes combined with separate treatment of concentrated waste streams (e.g., greywater, blackwater, kitchen waste). While this approach is increasingly seen as best practice, developing a first operational template and implementing it has come at a high cost for the utilities involved. The main challenges were organizational in nature. Utilities managing these systems suddenly had to respond to maintenance requests from individual customers, work across well-established government silos (water vs. sanitation vs. energy vs. waste), or actively find customers for their products (e.g., treated water, fertilizer, biogas).

These organizational challenges have proven much more difficult than expected. Often, legal and regulatory frameworks had to be actively modified, and new units or contractual agreements established within or across existing utilities [27]. In particular, public utilities starting to manage infrastructure that extends into private buildings have proven very complex and sensitive. The expansive negotiations involved in the innovation process have forced utilities to hire new staff with the necessary capabilities, especially skills to work effectively across silos. In addition, this example depended on developing an effective TIS structure that includes vacuum system suppliers, engineering consultants, technology providers, real estate developers, and communication experts [17].

(3) Bio-polymer extraction in centralized wastewater treatment plants (Zutphen and Epe, the Netherlands; Blackburn, the United Kingdom, and Campinas, Brazil)

A third example is conventional utilities developing new business lines through selling biopolymers recovered in centralized treatment plants. The wastewater treatment plants in Zutphen and Epe are illustrative examples. The respective utilities built up pioneering facilities that extract the biopolymer Kaumera from aerobic granular sludge generated in their wastewater treatment process [28]. Factors driving this development were mostly technical opportunities and spillovers from increasingly stringent emission reduction goals in the water, construction, and agricultural sectors. Kaumera is a versatile, water-absorbent biopolymer with applications across these sectors. It can be sold to the construction sector as a concrete binder, glue agent, and dye component, and can also be used in agriculture and horticulture as a biostimulant or seed protection agent. In addition to creating a new marketable product, Kaumera extraction also reduces costs and energy use for waste sludge disposal and processing by 25–30%, thus offering both environmental and economic benefits to the managing utility [28].

However, this approach also struggles with complex technical and social challenges. Technologically, it has proven very hard to produce a product with consistent properties, which has hampered market formation. Establishing a Kaumera business line at both plants was furthermore only possible by mobilizing an expansive network of funders and innovators and embarking on a multi-year innovation journey. The discovery of Kaumera originated from researchers at Delft University of Technology, who worked closely with utility experts in developing the technologies used in the first plants. Key partners in the innovation journey included engineering consultancies, biotechnology startups, as well as research foundations and national and EU-level institutional investors (see https://kaumera.com/en). Initially, the utility lacked critical expertise in biomaterial processing, market access, and regulatory compliance. These gaps were addressed mostly through collaboration with partners in the TIS structure described above. Market development strategies, including targeted customer engagement and establishing long-term sales contracts, helped secure stable revenue streams. As in the other cases, financial and regulatory hurdles have required proactive engagement with policymakers and investment in certifications to ensure compliance and market credibility [29]. Despite these efforts, long-term commercial success is still far from certain.

## 5 Supporting resource recovery transitions in the wastewater sector

We started this perspective by claiming that developing innovative resource recovery solutions from wastewater cannot stop short of identifying promising technologies. Instead, we showed that a socio-technical perspective is required, which asks how utilities must redefine themselves and their interactions with other organizations to seize these opportunities. Overall, we illustrated that these options provide promising innovation opportunities in specific places with particularly conducive context conditions, which may transform the sector's identity from managing waste to providing essential resources for a more sustainable future economy. However, tapping these potentials also needs some fundamentally new approaches in how new technologies are developed, managed, and scaled up by the main actors.

As we elaborated, the main tasks for those actors reside in strategically charting out this new opportunity space. First, it will take systematic effort to further assess the opportunities of specific resource streams that could be tapped. This requires engaging in the formation of new markets for the corresponding products (water provision, heat and power, chemicals, etc.), identifying key established players, competing products and services, their relative cost structures, and strategies for entering these markets. Or in other terms, utilities must master innovation in business models. Secondly, it will require utilities and other key actors in the sector to assess their current capabilities and identify instances where technological know-how, business activities, and organizational structures need to be actively adapted or expanded. A particularly important expertise is engaging in experiments with new approaches similar to the ones quickly highlighted above in order to gain a deeper understanding of the challenges and opportunities [30]. Finally, with increasing maturation, regulatory frameworks and utility mandates will need to evolve to enable scale up and to establish recovered resources as standard products. Utilities need to reflect whether they want to drive and own these processes from the beginning to full upscaling, whether they prefer to primarily act as incubators for new organizations, or whether they see their role in orchestrating broader innovation ecosystems that will subsequently bring these solutions to fruition.

All in all, we are aware that the sheer complexity of these tasks might easily overwhelm any single utility. Along the way, many hopes may prove to be hard to substantiate, markets may be less accommodating than hoped for, and competing against incumbents in the specific sectors may prove to be too hard for the limited resources that utilities can mobilize. But as the few examples above illustrated, we are confident that some of these activities will make a difference and ultimately change how society will look at wastewater and appreciate it increasingly as a resource trove instead of a mere inconvenience to get disposed of.

## CRediT authorship contribution statement

**Bernhard Truffer:** Writing – review & editing, Writing – original draft, Conceptualization. **Christian Binz:** Writing – review & editing, Writing – original draft, Conceptualization. **Eberhard Morgenroth:** Writing – review & editing, Writing – original draft, Conceptualization.

## Declaration of competing interest

The authors declare that they have no known competing financial interests or personal relationships that could have appeared to influence the work reported in this paper.
